# The Molecular Mechanisms of Mutations in Actin and Myosin that Cause Inherited Myopathy

**DOI:** 10.3390/ijms19072020

**Published:** 2018-07-11

**Authors:** Steven Marston

**Affiliations:** National Heart and Lung Institute, Imperial College London, London W12 0NN, UK; s.marston@imperial.ac.uk; Tel.: +44-020-7594-2732 or +44-07484-330098

**Keywords:** actin, myosin, myopathy, mutation, contractility, Ca2+-regulation, A-triad, super relaxed state, myosin mesa

## Abstract

The discovery that mutations in myosin and actin genes, together with mutations in the other components of the muscle sarcomere, are responsible for a range of inherited muscle diseases (myopathies) has revolutionized the study of muscle, converting it from a subject of basic science to a relevant subject for clinical study and has been responsible for a great increase of interest in muscle studies. Myopathies are linked to mutations in five of the myosin heavy chain genes, three of the myosin light chain genes, and three of the actin genes. This review aims to determine to what extent we can explain disease phenotype from the mutant genotype. To optimise our chances of finding the right mechanism we must study a myopathy where there are a large number of different mutations that cause a common phenotype and so are likely to have a common mechanism: a corollary to this criterion is that if any mutation causes the disease phenotype but does not correspond to the proposed mechanism, then the whole mechanism is suspect. Using these criteria, we consider two cases where plausible genotype-phenotype mechanisms have been proposed: the actin “A-triad” and the myosin “mesa/IHD” models.

## 1. Introduction

The discovery that mutations in myosin and actin genes, together with mutations in the other components of the muscle sarcomere, are responsible for a range of inherited muscle diseases (myopathies) has revolutionised the study of muscle, converting it from a subject of basic science to a relevant subject for clinical study and has been responsible for a great increase of interest in muscle studies.

We now know that myopathies are linked to mutations in five of the myosin heavy chain genes, three of the myosin light chain genes, and three of the actin genes (Table 1). One of the reasons for rapid progress in the early days of gene sequencing is the simplicity of their genetics. Both myosin and actin are highly conserved proteins, their genes are relatively small, and exon-swapping isoforms are minimal in myosin heavy chain and absent in myosin light chains and actin (compare this with the complexity of tropomyosin, another highly conserved muscle protein [1]). Variants of these proteins are tissue and developmentally specific and are usually transcribed from separate genes [2,3,4,5]. There are few polymorphisms in the actin and myosin genes; this means that almost any mutation in one of these genes is likely to have a functional effect that would be manifested as a clinically relevant disorder. Since there is a wealth of structural and functional knowledge of actin and myosin one would expect that it is possible to explain the dysfunction caused by a mutation at the molecular level. This review aims to determine to what extent we can explain disease phenotype from the mutant genotype.

## 2. From Clinic to Fundamental Studies

In practice, the situation is far more complex than a simple pathway of one mutation causing a specific abnormality that causes a specific disease. This has been apparent from the start of investigations where the same disease phenotype could be caused by many mutations in several different genes, for instance over 1500 mutations in nine genes have been linked with the single phenotype of hypertrophic cardiomyopathy [6]. Conversely different mutations in one gene can lead to different diseases, for instance *MYH7* mutations have been linked to hypertrophic cardiomyopathy, dilated cardiomyopathy, Laing distal myopathy and Scapuloperineal/limb girdle syndromes (Table 1, [3]). Thus, there are likely to be a variety of initial molecular abnormalities to be discovered and the way these convert into the disease phenotype may involve multiple pathways.

The clinical picture, on which any study of the genotype-phenotype relationship is based, is usually not very clear. It should be understood that the way in which myopathies are discovered starts with a clinical diagnosis combined with a demonstration of a linked genetic abnormality. Both these processes are subject to mistakes or inadequate investigations but the quality of this data is rarely considered by basic scientists. A full family history is only performed in a minority of cases yet this is the only way that the genetic linkage can be definitively proved. In fact, a significant proportion of myopathies are thought to be due to de novo mutations in the proband only, especially in the congenital skeletal muscle myopathies [9,10]. The quality and quantity of clinical data is dependent on the medical specialty, thus heart diseases are intensively studied and well-funded with a plethora of diagnostic techniques whilst skeletal muscle myopathies are studied by a much smaller group of practitioners [4,11,12,13]. A significant proportion of myopathies can be shown to be inherited and yet the causative mutated gene cannot be found; this is most likely because the phenotype is caused by simultaneous mutations in more than one gene. At the moment it is only feasible to study diseases caused by a single mutation. 

Actin and myosin are of such fundamental importance that most mutations are likely to be lethal and so are never seen in the patient population. The myopathies we observe are mostly quite mild and are disabling rather than lethal. Skeletal muscle myopathies, such as nemaline myopathy, are congenital and may lead to death at a young age but cardiomyopathies are usually not manifested until puberty, when a growth spurt puts excessive strain on the heart. Thus, the molecular changes due to myopathy mutations are likely to be quite small, making investigation difficult. Moreover, the disease seen in the cardiac patient when they get to the clinic is likely to be in an advanced stage so that secondary pathologies consequent upon the initial constitutive triggering abnormality may dominate the clinical phenotype and this further complicates determining what the initial abnormality is. Finally, the majority of myopathy-causing mutations are heterozygous, indicating one normal and one mutant allele; the extent to which the abnormality is manifested is rather variable and a significant proportion of patients carrying the mutation never show the symptoms of myopathy [14]. This variable penetrance is itself largely due to a variable genetic background with some patients having compensatory genetic or epigenetic characteristics, as has been shown in mouse models [15,16].

The most thoroughly studied inherited myopathies are the cardiomyopathies, particularly hypertrophic cardiomyopathy (HCM) and dilated cardiomyopathy (DCM) [8,10,11,12,13,14]. DCM accounts for 30–40% of heart failure and is the second commonest cause after coronary artery disease. The disease is characterised by left ventricle dilation and impaired systolic function and is believed to be caused by mutations in up to 50% of cases. Mutations in around 50 genes have been linked with familial DCM, including all proteins of the contractile apparatus. Truncating mutations in the *TTN* gene account for 20% of fDCM and mutations in thin filament proteins make up to 11% of cases. Hypertrophic cardiomyopathy (HCM) is a primary disease of cardiac muscle clinically defined by a hypertrophied, non-dilated left ventricle in the absence of any other aetiology. It is a common disorder affecting one in 500 of the population and is the leading cause of sudden cardiac death in young individuals. HCM is primarily caused by monogenic mutations in sarcomeric proteins, notably myosin heavy chain, (*MYH7* gene) and cardiac myosin binding protein C (MyBP-C, *MYBPC3* gene) which each make up about 40% of known mutations. MyBP-C is associated with the muscle thick filament; it binds to thick filaments through interactions with LMM, S-2 and myosin light chain and also binds to actin.

## 3. How Mutations Cause Abnormal Function

A mutation may have a variety of fundamental mechanisms. Most amenable to investigation is the poison peptide mechanism. The mutated gene is normally transcribed so that the abnormal protein is produced and incorporated into the sarcomere and leads to abnormal function. In principle, heterozygous individuals should express equal quantities of the poison peptide and normal protein. This is a hard thing to measure in most instances although charge-change mutations in actin have been successfully identified by 2D electrophoresis or mass spectrometry and they usually yield a proportion less than 50% [17,18,19,20].

If a mutation introduces a chain terminating codon or disrupts an exon-intron boundary a truncated protein may be expressed. In practice the truncated protein is never observed in the patient tissue samples. Cellular maintenance mechanisms remove the abnormal protein either at the transcription stage (nonsense-mediated RNA decay), or by targeting the truncated peptide for destruction via the ubiquitin-proteasome pathway. This leads to a deficiency of the protein in question and this deficiency may be sufficient to trigger the myopathy. Truncating mutations in the *MYBPC3* gene that codes for cardiac MyBP-C are associated with hypertrophic cardiomyopathy and are one of the most thoroughly investigated examples of haploinsufficiency [21,22]. Haploinsufficiency has never been observed in any actin or myosin mutations, presumably because this is incompatible with survival.

In rare cases the gene product may be completely absent but the patient survives due to substitution of a different gene. This has been well documented for the *ACTA1* gene. Cases of nemaline myopathy children have been found with a homozygous *ACTA1* null mutation that express no skeletal actin in their muscles. In this case cardiac actin (*ACTC* gene) that is normally expressed in foetal skeletal muscle is expressed in its place. This type of substitution is only possible due to the high homology between skeletal and cardiac actin [23,24].

## 4. How We Can Investigate Mutations That Cause Myopathies

To identify how a mutation can cause an abnormality we need to investigate structure and function of the mutated protein. We are fortunate that, structurally, actin and myosin have been investigated in detail for a long time by X-ray crystallography, electron microscopy, and other techniques with ever increasing resolution, however, we do not have precise structures for every functional state. In muscle both actin and myosin are formed into filaments that are not amenable to high resolution methods. The highest resolution is obtained in monomeric proteins which may not have the same structure and certainly do not have the same function as the thick and thin filaments. In addition, structural studies only show a static snapshot of structure whilst the contracting muscle is an extremely dynamic and cooperative/allosteric ensemble of proteins. Nevertheless, it is possible to pinpoint mutations in these structures and to propose hypotheses as to how these would alter structure and hence function. 

Functional studies are more difficult: it is usual to use site-directed mutagenesis of pure proteins for structure-function investigations. This is particularly difficult for both actin and myosin where the complex folding cannot be achieved in bacterial expression systems. Human sequence actin has been successfully expressed in functional form in baculovirus/sf9 systems, however there are few studies of myopathy mutations [25,26,27,28,29]. Recently myosin heavy chain has been successfully expressed in small quantities in C2C12 myoblasts and the effects of mutation has been studied [30,31]. Transgenic and knock-in mouse models have been extensively used for actin [18,32,33,34,35], myosin heavy chain [36,37] and myosin light chain mutation studies [38,39,40]. Actin has the same sequence in man and mouse and light chain sequences are similar but mouse heart predominantly expressed α-myosin heavy chain (*MYH6* gene) rather than β-myosin (*MYH7*) which predominates in human heart so the mutations have to be studied in an α-myosin heavy chain background which may significantly impact upon the effects of the mutation [41]. The vast majority of such studies have been devoted to HCM mutations with a small number of models having DCM or nemaline myopathy-causing mutations. A few functional studies have used human tissue: most skeletal myopathy patients have muscle biopsies as part of their diagnosis and these have been a source of up to 10mg tissue for study [42,43,44]. A significant number of HCM patients have a septal myectomy operation to relieve outflow tract obstruction and this can yield a few grams of mutant tissue [45,46]; finally, explanted hearts from DCM patients with identified causative mutations undergoing heart transplant provides a rare source of tissue for investigation [47,48]. Since this tissue is stored frozen the type of studies that can be done with such samples is limited.

The objective is to use these systems to find the molecular mechanism alterations caused by the mutation to explain the disease. However, both myosin and actin are multifunctional proteins and mutations that affect different functional regions are likely to have different effects. The functions affected by mutations can be divided into

- Thick and thin filament assembly from monomers
- Force transmission from cross bridge to Z-band
- Force generation by contractile motor function
- Regulation of contractility

Methodology for investigating filament assembly is much less well developed than our ability to study the acto-myosin motor or its regulation by Ca^2+^ and phosphorylation.

## 5. From a Molecular Phenotype to an Explanation of Disease

Despite a large number of studies over the last 20 years, most attempts to positively link a mutation to a functional disease-causing change have had limited success. There are two problems here. Firstly, the small differences caused by the mutations, thus a sensitive signal is required. The most-used indicator of mutational effects is a shift in the myofilament Ca^2+^-sensitivity. This is a useful readout because it could be affected by many different changes in motor and regulatory function, however it is also non-specific: Ca^2+^-sensitivity is merely a measure of the equilibrium between active and relaxed cross bridges which could be altered by multiple causes [49]. In a recent survey it was shown that mutations, whether in skeletal or cardiac muscle genes tend to give changes of no more than 2-fold in Ca^2+^-sensitivity. The implication of this seems to be that large changes are incompatible with survival. The second problem is that most of the muscle diseases in Table 1 are quite rare and trying to elucidate a mechanism that can predict a consistent disease-specific behaviour in other mutations with a small sample size is fraught with difficulty especially if functional information is limited. There are numerous examples of hypotheses based on small samples and incomplete experimentation that proved to be wrong.

### 5.1. MYH7 R403Q HCM Mutation Reduces Contractility

As the first mutation demonstrated to be associated with HCM the *MYH7* R403Q mutation has been studied more than any other myosin mutation. Early studies of the R403Q mutation created some confusion since study of the mutation in a skeletal muscle biopsy and expression of the mutation in Baculovirus and in Dictyostelium expression systems indicated a substantially lower rate of crossbridge turnover [50,51,52], whilst a human heart biopsy with this mutation and a transgenic mouse with the mutation introduced into *MYH6* showed an increase in crossbridge turnover rate [53,54,55]. Some elaborate but ultimately unconvincing, models were proposed for how a loss of function could cause HCM and yet be different from the loss of function that causes DCM. Most published studies addressed the effect of myosin heavy chain mutations on motor functions [56], and only a few studies measured myofibrillar Ca^2+^-sensitivity and these generally show enhanced myofibrillar Ca^2+^-sensitivity [37,57]. The current consensus is that despite the early work, *MYH7* R403Q mutation produces a hypercontractile phenotype with enhanced Ca^2+^-sensitivity in common with other HCM-linked mutations.

### 5.2. MYBPC3 HCM Mutations Are Mild and Late Onset

In the earliest studies data suggested that *MYBPC3* mutations may be the pre-dominant genetic substrate for HCM in elderly patients, among whom the natural history is generally favourable but this was based on a small and, in retrospect, selective sample [58,59]. When a large cohort (71 unrelated *MYBPC3* mutants) was investigated, patients with *MYBPC3* mutations did not differ significantly from patients with thick filament-HCM, thin filament-HCM, or genotype-negative HCM with respect to age at diagnosis, degree of hypertrophy, incidence of myectomy, or family history of HCM or sudden death [60].

### 5.3. DCM Mutations Cause a Decrease in Ca^2+^-Sensitivity

As with HCM, the thin filament protein mutations associated with DCM have been extensively investigated using recombinant mutant proteins [61,62]. Early studies have shown a common molecular phenotype: DCM mutations cause a lower Ca^2+^-sensitivity, correlated with lower Ca^2+^-affinity for TnC [63], a lower rate of crossbridge turnover and sometimes, lower cooperativity. The initial conclusion, therefore, was that DCM mutations produce a hypocontractile molecular phenotype (the opposite of HCM mutations) [64,65]. However, further studies of DCM mutations with proteins from human or transgenic mouse heart tissue show that the real situation is more complex than indicated with pure recombinant proteins. Ca^2+^-sensitivity is both increased and deceased by DCM mutations, depending on the experimental conditions. A literature survey in 2011 found in six out of 14 mutations, Ca^2+^-sensitivity was not decreased by the DCM mutation [19] and Table 2 shows how little Ca^2+^ sensitivity and the DCM phenotype correlate even for the same mutation in different measuring systems (decreased Ca^2+^-sensitivity corresponds to an increased ratio in this table). Subsequently, the blunting of the relationship between troponin I phosphorylation and Ca^2+^-sensitivity modulation has emerged as the key factor in determining the phenotype [61,66]. 

### 5.4. Skeletal Muscle Myopathy Phenotype due to ACTA1 Mutations Can Be Predicted from Actin’s Structure

Mutations in the skeletal muscle actin gene, *ACTA1* are responsible for up to 20% of congenital myopathies with a variety of pathologies that includes nemaline myopathy, intranuclear rod myopathy, actin myopathy, and congenital fibre type disproportion [4]. In their review of 2003, Sparrow et al. considered how these actin mutations might affect muscle function at the molecular level and thus cause the disease [72], however, investigations of genotype–phenotype relationships experimentally did not support the predictions. In Feng’s review [73] thirty congenital myopathy-causing *ACTA1* mutations were analysed using a range of biochemical and in vitro approaches. In some cases structural predictions are a good indicator of the likely molecular defects of actin mutations; for example, 2/3 of the predicted polymerisation defects were confirmed in functional studies. However, in many cases there is disagreement between the structure and functions of mutants. Only three out of 11 predicted folding defects were confirmed, and half the mutations that are close to the actin nuclear export signal resulted in cytoplasmic aggregates rather than intranuclear rods. None of the three mutations causing altered binding affinity for alpha-actinin are located at known contacts between alpha-actinin and actin. A few mutations resulted in unfolded or unstable actins, some led to reduced polymerisation capacity, formation of aggregates in cells, altered expression of other sarcomeric proteins, impaired motility, and contractility. The general conclusion was that we have insufficient data, both functional and genetic to predict any skeletal muscle actinopathy from structure.

For the purposes of this review we should only consider mutations in actin and myosin that have an optimum chance of yielding a correct mechanism. Our detailed knowledge of the structures of actin and myosin in several functional states and their interactions with other proteins (MyBP-C, troponin, tropomyosin etc.) is a necessary stating point, but it is not enough. To optimise our chances of finding the right mechanism we must study a myopathy where there are a large number of different mutations that cause a common phenotype and so are likely to have a common mechanism: a corollary to this criterion is that if any mutation causes the disease phenotype but does not correspond to the proposed mechanism, then the whole mechanism is suspect.

A second important criterion is that gain-of-function mutations are much easier to study than loss of function mutations. The reason for this is simply that to obtain a gain of function, particularly with many different mutations, implies a specific mechanism whilst a loss of function could occur by an unlimited range of mechanisms. Using these criteria we can consider two cases where plausible genotype-phenotype mechanisms have been proposed: the actin “A-triad” and the myosin “mesa/IHD” models.

## 6. A Triad System

Although studies of the functional mechanism of actin mutations has been frustrated by the multifunctional nature of actin there is one case where this has been turned to advantage. The interaction of actin with tropomyosin is very specific and fundamental to muscle regulation; consequently, it has been extensively investigated. The role of tropomyosin has been evident since the concept of the steric blocking mechanism was established and has since been developed into comprehensive functional [74] and structural [75,76] models that have stood the test of time [77].

A few actin mutations specifically affect this interaction. This was first demonstrated for the E93K mutation in drosophila *Act88F* gene [78,79]. This is a flightless, loss of function mutation. In vitro it was clearly demonstrated that actin behaviour is normal but in the presence of tropomyosin the contractile activity, as measured by in vitro motility assay is switched off. The interpretation was that in some way this charge change caused the nearby tropomyosin strand to move to a more blocking position than normal. That this was simply a shift of equilibrium effect could be demonstrated by the restoration of contractile function by adding troponin +Ca^2+^ or NEM-S-1 (*N*-ethyl maleimide-treated myosin subfragment-1 is locked in the rigor state independent of ATP), both agents that switch tropomyosin from the blocked to the closed and open states.

The first human actin mutation that had a comparable effect was discovered in a patient with congenital fibre-type disproportion. In a biopsy from this patient with a heterozygous *ACTA1* D292V mutation it was observed that actin function was normal but the motility was switched off by tropomyosin and in this case could not be restored by NEM S-1 or troponin +Ca^2+^ [17].

**Figure 1:**
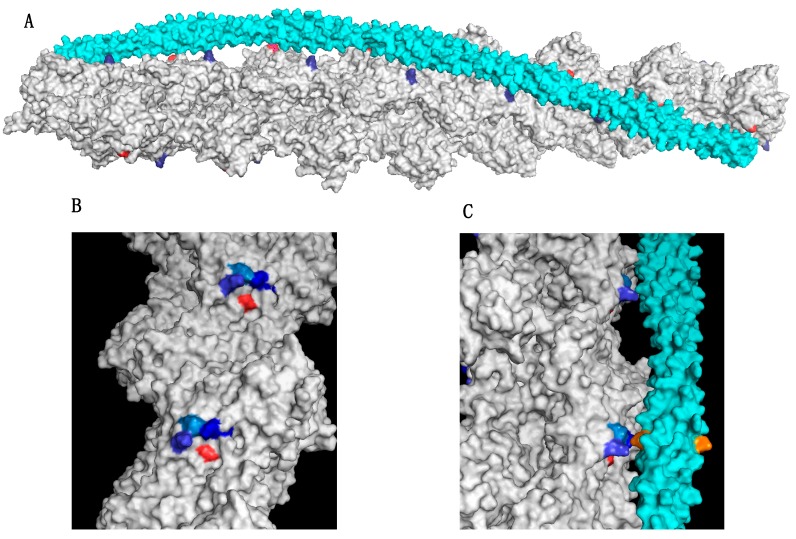
The actin-triad motif (**A**) Structure of the actin-tropomyosin interface showing one of the two tropomyosin molecules (cyan) bound to the actin double helix (grey). Surface rendering using PyMol with coordinates from Li et al. [80]. Actin Asp25 is coloured red and Lys 326 in the A-triad is coloured blue. (**B**) Location of actin triad residues Lys326, Lys328, and Arg147 in blue and Asp272, red. (**C**). Side view showing the interaction of tropomyosin (cyan) with two successive A-triads. The cognate amino acid in tropomyosin that interacts with the triad (E139) is shown in orange. Figures based on refs [81,82].

The structural basis for the effects of mutations on the blocked-closed-open equilibrium was provided by the structural analysis of the Lehman group. The initial study was by Li et al. [80] and has since been refined but not fundamentally altered [76]. This study identified the points on actin that interact with tropomyosin. Asp25 and the triad K326, K328, and R147 make electrostatic interactions with tropomyosin that stabilise the closed/blocked state. A corresponding set of acidic residues in tropomyosin in each of the 7 repeats of tropomyosin interact with the actin A-triad sites. (see Figure 1). The functional relevance of these interactions for controlling filament activity was demonstrated with a series of tropomyosin mutations that were found to cause a range of gain-of-function phenotypes: distal arthrogryposis, camphylodacty and a more extreme ‘stiff patient’ syndrome. Every one of nine gain of function tropomyosin mutations described in two studies caused an increase in myofilament Ca^2+^-sensitivity in vitro and involved destabilisation of the interaction either with actin Asp25 or the A-triad [81,82]. A number of predictions follow from these finding, in particular that charge change mutations on actin or tropomyosin in the interaction sites between actin and tropomyosin at these two points would destabilise the blocked-closed structure and cause a gain of function and that a mutation that stabilised the blocked-closed structure would cause a loss of function.

These predictions explain the action of two previously investigated pathological actin mutations. The “stiff child” syndrome is characterised by generalised skeletal muscle hypercontraction. The first such patient diagnosed was found to have a mutation in *ACTA1* K326N and has been shown to result in an increased myofilament Ca^2+^-sensitivity [44]. This mutation causes a charge change in one of the actin-triad amino acids and on the basis of this model is expected to be hypercontractile. The second actin mutation is D292V, already mentioned [17,81]. D292 is located very close to the A-triad and is oppositely charged to the three basic amino acids of the triad proposed to stabilise the closed/blocked state (Figure 1). It has been proposed that D292 modulates or ‘steers’ the triad interaction with tropomyosin so that the closed/blocked state is not too stable to allow for changes in tropomyosin position during muscle regulation. The D292V mutation would then cause the tropomyosin interaction with the triad to become stronger and thus lock tropomyosin in the closed/blocked state. [81,83].

This hypothesis is backed up by computational chemistry. The interaction of α-tropomyosin with F-actin was examined by optimizing the energy of the complex for a wide range of tropomyosin positions on F-actin. The resulting energy landscape provides a full-map of the F-actin surface preferred by tropomyosin, revealing a broad energy basin associated with the tropomyosin position that blocks myosin-binding. The gain of function mutations in both actin and tropomyosin cause the energy minimum to become shallower and to shift toward the open state position whilst the D292V mutation made the energy minimum deeper [83,84,85]. Subsequently another mutation, this time in the cardiac actin gene: *ACTC* A295S is associated with hypertrophic cardiomyopathy and is located at the base of, and just behind, the actin A-triad (close to D292) [86]. The functional effects were studied by introducing the mutation into *Drosophila* actin. It was shown to cause a gain of function by enhancing Ca^2+^-sensitivity and also destabilising the A-triad-tropomyosin interaction [87]. Thus the modification of the charged interactions at the actin A-triad-tropomyosin (and Asp25-tropomyosin) interfaces currently satisfies our criteria for a plausible model of a mechanism for both gain-of-function and loss-of-function actin mutations at the atomic level to modulate the thin filament switch.

## 7. The Myosin Mesa and Interacting Heads Motif Model of HCM

HCM mutations in myosin present a particularly interesting case since several hundred mutations have been found that produce the same phenotype; thus the search for finding what these mutations have in common should be easier. The essential primary characteristic of HCM at the myofilament level is that the mutations cause a hypercontractile phenotype [88]. Myofibrillar Ca^2+^-sensitivity increased 2-fold and muscle output increased substantially [89,90]. Power and work output are higher than normal but efficiency is correspondingly reduced [32,91]. It has been hypothesised that the impaired energetics accompanying reduced efficiency triggers the secondary changes that constitute the disease phenotype [92,93]. 

How could so many different mutations produce the same phenotype? Traditionally mutations have been studied individually and the effects of the mutations interpreted in terms of the known and proposed molecular transitions involved in the motor function of myosin. This has not been successful as noted above and it is not surprising, since it is not reasonable to expect mutations to cause a range of abnormalities and produce the same phenotype. Researchers had not looked for what the common mechanism should involve. Contractile force in muscle is dependent not only on the individual properties of each cross-bridge, but the availability of cross-bridges to participate in actin-myosin interactions. Thin filament regulation is based on this principle: Ca^2+^, through the cooperative-allosteric troponin-tropomyosin switch controls the availability of actin to interact with myosin heads [94,95]. Could there be an equivalent regulatory mechanism in the thick filament [96]?

**Figure 2:**
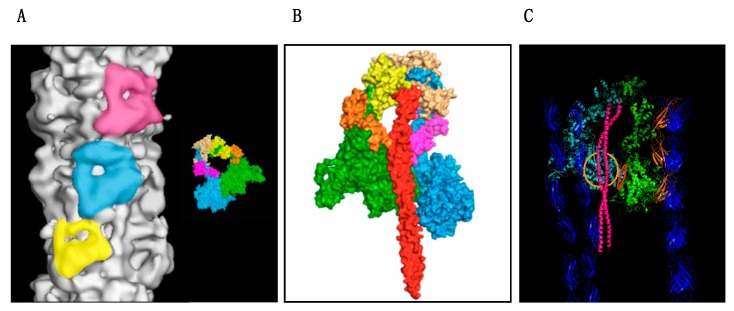
The interacting heads motif on the myosin filament. Figures based on the atomic model of the human cardiac muscle myosin filament made by Al-Khayat et al. [103]. (**A**) Three successive IHM are highlighted on the surface of the thick filament. The inset shows the comparable high resolution structure of the smooth muscle IHM [101]: MHC “free”’ head is blue and ‘docked’ head is green. Light chains are brown, orange yellow and pink. (**B**) Fitted coordinates of the myosin heads and subfragment-2 (S2) (red) viewed from inside the filament showing how the proximal S-2 interfaces with the folded back myosin mesa of the “docked” head and the converter domain of the “free head”. (**C**) Fitted model incorporating titin, blue, and MyBP-C, orange into the structure of Figure 2B. MyBP-C appears to contact the mesa and light chains of the “free” head. The myosin mesa of the ‘docked head is shown with the orange circle and the converter domain-“docked” head interface is shown in red.

The breakthrough here came in an indirect way; An examination of the known structures of myosin heads (S-1) showed that there is a relatively flat surface on the ‘top’ of S-1 that has not been implicated in any of the known motor interactions: actin binding, ATP binding or the conformational change that moves the lever arm, yet this surface contains a concentration of basic amino acids suggesting it should be forming an interface with another protein. The most critical finding was that the surface—termed the “myosin mesa”—was a hot-spot for charge change mutations associated with HCM and it was hypothesised that these mutations would destabilise the interface [97,98]. It was soon realised that the main interface of the myosin mesa was with myosin itself since myosin can pack down onto the surface of the thick filaments forming the “interacting heads motif” (IHM), where it is unavailable to interact with actin (Figure 2). Thus the idea was proposed that HCM mutations could destabilise the IHM and release heads for interaction with actin [99,100].

### The Interacting Heads Motif (IHM)

Current thinking about the actin–myosin interaction is that the thick filament exists in two states; these have for a long time been described as ordered and disordered thick filament states and their transition appeared to be regulated by phosphorylation. The “ordered” state corresponds to a structure in which the myosin heads, instead of pointing away from the filament towards the actin are folded backwards and inwards to form the compact IHM close to the thick filament shaft (Figure 2). The folded structure was first described in smooth muscle myosin when unphosphorylated [101]. Smooth muscle is switched on by Ca^2+^-dependent phosphorylation of the myosin regulatory light chain which releases the myosin heads from the IHM allowing actin myosin interactions. The same IHM motif has been seen in every muscle examined including human cardiac muscle [102,103,104] suggesting a highly conserved and functionally relevant structure. 

Vertebrate striated muscle, skeletal, and cardiac are regulated by Ca^2+^ acting on troponin rather than the thick filament, but the regulatory function of the IHM is retained in part. Physiologists have pointed out that relaxed striated muscle consumes virtually no energy whilst actomyosin systems in vitro only regulate at best over a 20-fold range. It was argued that this was because isolated myosin was damaged in some way, however it could equally be because there is a second, “super-relaxed” state of myosin, corresponding to the IHM, that can only be achieved in an intact thick filament. The presence of a super-relaxed state in striated muscle including human cardiac muscle has now been demonstrated, using single ATP turnover kinetics of relaxed skinned muscle which shows a fast and a slow process with t_1/2_ of 14.3 and 224 secs respectively in human cardiac muscle. In normal cardiac muscle the proportion of heads in the “super-relaxed state” was 27.6 ± 0.7% [105,106,107]. The IHM probably involves additional proteins apart from myosin: MyBP-C is one of the most likely participants; cardiac MyBP-C is an extended protein consisting of 10 domains and it has been established that N-terminal domains C0-C2 lie over the myosin mesa and also contact the proximal S-2 and MLC2 (Figure 2). Furthermore, this is a regulated structure with IHM stability being modified by MLC2 and MyBP-C phosphorylation [99,108,109,110]. 

## 8. Unifying Hypothesis for Myosin and MyBP-C mutations Causing HCM

The association of HCM mutations with the IHM is now very strong. Homburger’s analysis [97] of a large dataset of HCM mutations found three hotspots: the myosin mesa, the proximal S-2 that interfaces with the myosin mesa and the converter domain. Importantly the convertor domain mutations were all clustered at the surface where the “docked head” of the IHM contacts the convertor domain of the “free head”. 70% of myosin variants map to the myosin mesa and 100% of those that map to the convertor domain are disease-producing. Only 20% of HCM mutations remain unaccounted for on the basis of direct contacts in the IHM, but these could well affect IHM stability indirectly. Alamo et al. [111] covered an even larger cohort of HCM patients and came to a similar conclusion. Analysis of 6112 HCM variants indicated that the 72% of mutations that changed electrostatic charges disproportionately altered IHM interaction residues (expected for random distribution 23%; found with HCM mutations 54%, *p* = 2.6 × 10^−19^, indicating a highly significant association of HCM mutations with the IHM [111]). The correlation appears to hold good for the other proteins also: all 4 ELC and RLC HCM mutations examined by Alamo et al. would destabilise the IHM and it has been experimentally demonstrated that ablation of MyBP-C destabilises the super-relaxed state and that HCM mutations in MyBP-C that promote haploinsufficiency also destabilise the super-relaxed state [105] by reducing the fraction of super-relaxed heads from 27.6% in wild-type to 22.3 ± 1% in a MYBPC3 mutation accompanied by a shortening of the lifetime of the super-relaxed state from 224 to 160 s, indicating destabilisation of the IHM. 

At present, the IHM and the super-relaxed state have only been directly connected for the R403Q myosin mutation [112], but the weight of indirect evidence is compelling. The correlation of mutations with potential to destabilise the IHM seems solid for myosin heavy chain mutations and also for myosin light chain mutations and MyBP-C mutations causing haploinsufficiency. The effects of destabilising the IHM would be incomplete relaxation and more heads available leading to hypercontractility—the key abnormalities of HCM. The higher proportion of myosin heads would also act upon the thin filaments cooperatively to promote the on state, which would cause an increase in the apparent Ca^2+^-sensitivity of muscle activation as observed. Finally, the pathological mechanism of thick and thin filament mutations causing HCM can be unified since both will tend to increase the interactions between myosin heads and actin.

A final piece of evidence to support the IHM hypothesis is the development of an anti HCM drug MYK-461 (now known as Mavacampten), found by high throughput screening. It has been shown to be effective both in vitro and in vivo in reversing most of the basic symptoms of HCM [113,114]. A recent study has shown that the mode of action of Mavacampten is to stabilise the IHM structure and enhance the super-relaxed state of myosin [112].

## Figures and Tables

**Table 1:** Myosin and actin genes, the protein expressed, its tissue specificity, and number disease-causing mutations reported (early 2017). The table is compiled from data in references [3,4,7,8].

Protein	Gene	Protein Name	Predominant Expression in Muscle	Myopathy	Number of Mutations
Myosin heavy chain	*MYH1*	MyHC-2X	Fast-twitch skeletal muscle (Type IIx)		
	*MYH2*	MyHC-2A	Fast-twitch skeletal muscle (Type IIx/IIa)	“myopathy”, inclusion body myopathy, distal and proximal myopathy, opthalmoplegia	15
	*MYH3*	MyHC-embryonic	Embryo	Distal arthrogryposis types 1, 2A, 2B, 8, Freeman-Sheldon, Sheldon-Hall syndrome	33
	*MYH4*	MyHC-2B	Fast-twitch skeletal muscle (Type IIb)		0
	*MYH6*	α-MyHC	Atria	Hypertrophic cardiomyopathy, dilated cardiomyopathy, atrial-septal defect, other congenital defects	33
	*MYH7*	β-MyHC	Cardiac ventricles; slow-twitch skeletal muscle (Type I)	Hypertrophic cardiomyopathy, Dilated cardiomyopathy, left ventricular non-compaction, Laing distal myopathy, Scapuloperineal and limb girdle syndromes	>800
	*MYH8*	MyHC-perinatal	Fetal skeletal muscle	Distal arthrogryposis DA7	1
Essential light chain (Alkaline light chain)	*MYL1*	MLC1f, MLC3f	Fast-twitch skeletal muscle		
	*MYL3*	VLC1, MLC1V	Cardiac ventricles; slow-twitch skeletal muscle	Hypertrophic cardiomyopathy, Dilated cardiomyopathy	21
	*MYL4*	ALC1	Atria; embryonic cardiac ventricles and skeletal muscle	Atrial fibrillation	1
Regulatory light chain	*MYL2*	MLC-2	Heart; skeletal muscle	Hypertrophic cardiomyopathy	24
	*MYL7*	MYL2A, MLC-2a	Atrial; embryo		0
	*MYLPF*	MLC2B, MLC-2f	Fast-twitch skeletal muscle		0
Actin	*ACTA1*	Skeletal actin	Skeletal muscle	Nemaline myopathy, actin myopathy, congenital fibre-type disproportion, stiff patient	235
	*ACTC*	Cardiac actin	Cardiac muscle, embryonic skeletal muscle	Hypertrophic cardiomyopathy, dilated cardiomyopathy, left ventricular non-compaction, atrial-septal defect	40
	*ACTA2*	Smooth muscle actin	Vascular smooth muscle	Aneurism	1

**Table 2:** Variability of the effect of DCM-causing mutations on Ca^2+^ sensitivity in native and synthetic thin filaments.

Mutation	Ratio EC_50_ mut/wt for Native Thin Filaments	Ref.	Ratio EC_50_ mut/wt for Synthetic System	Ref.
*TNNT2* DK210 (homozygous)	1.6	[67]	0.65 Recombinant troponin, Asα-tropomyosin	[62]
	2.2	[61]		
*TNNC1* G159D	0.55	[68]	1.8 Recombinant troponin, ASα-tropomyosin	[62]
			0.99 Mouse heart fibres exchanged with recombinant TnC	[69]
			1.9 Human heart troponin, ASα-tropomyosin	[68]
*TPM1* E54K	1.0	[61]	1.7 Recombinant troponin, ASα-tropomyosin	[62]
			0.52 Recombinant troponin, ASα-tropomyosin	[63]
*TPM1* D230N	0.43	[61]	1.7 Recombinant troponin, ASα-tropomyosin	[70]
			1.7 Skeletal muscle troponin, native αtropomyosin	[61]
*ACTC* E361G	0.95	[33]	3.3 Skeletal muscle troponin, native α-tropomyosin	[33]
			0.45 cardiac myofibrils	[71]

The effect of a mutation on the Ca^2+^-sensitivity of myofilaments is expressed as the ratio EC_50_ mut/wt > 1 means reduced Ca sensitivity, ratio EC_50_ mut/wt < 1 means increased Ca^2+^ sensitivity. ASα-tropomyosin is recombinant α-tropomyosin (Tpm 1.1) with Ala-Ser in place of native acetylated N-terminus. Table is reproduced with modifications from Memo et al., 2014 [61]. *TNNT2*; gene for troponin T, *TNNC1*; gene for troponin C, *TPM1*; gene for tropomyosin Tpm1.1, *ACTC*; gene for cardiac actin.
